# Developmental Fluoxetine Exposure Alters Behavior and Neuropeptide Receptors in the Prairie Vole

**DOI:** 10.3389/fnbeh.2020.584731

**Published:** 2020-11-16

**Authors:** Rebecca H. Lawrence, Michelle C. Palumbo, Sara M. Freeman, Caleigh D. Guoynes, Karen L. Bales

**Affiliations:** ^1^Department of Psychology, University of California, Davis, Davis, CA, United States; ^2^California National Primate Research Center, University of California, Davis, Davis, CA, United States; ^3^Department of Behavioral Neuroscience, Oregon Health & Science University, Portland, OR, United States; ^4^Department of Biology, Utah State University, Logan, UT, United States; ^5^Department of Psychology, University of Wisconsin, Madison, WI, United States; ^6^Department of Neurobiology, Physiology and Behavior, University of California, Davis, Davis, CA, United States

**Keywords:** oxytocin receptor, vasopressin receptor, serotonin receptor, 5-HT, autism, antidepressant, SSRI, autoradiography

## Abstract

Developmental exposure to selective serotonin reuptake inhibitor (SSRI) increases the risk of Autism Spectrum Disorder (ASD), however, the underlying neurobiology of this effect is not fully understood. Here we used the socially monogamous prairie vole as a translational model of developmental SSRI exposure. Paired female prairie voles (*n* = 20) were treated with 5 mg/kg subcutaneous fluoxetine (FLX) or saline (SAL) daily from birth of the second litter until the day of birth of the 4th litter. This design created three cohorts of FLX exposure: postnatal exposure in litter 2, both prenatal and postnatal exposure in litter 3, and prenatal exposure in litter 4. Post-weaning, subjects underwent behavioral testing to detect changes in sociality, repetitive behavior, pair-bond formation, and anxiety-like behavior. Quantitative receptor autoradiography was performed for oxytocin, vasopressin 1a, and serotonin 1a receptor density in a subset of brains. We observed increased anxiety-like behavior and reduced sociality in developmentally FLX exposed adults. FLX exposure decreased oxytocin receptor binding in the nucleus accumbens core and central amygdala, and vasopressin 1a receptor binding in the medial amygdala. FLX exposure did not affect serotonin 1A receptor binding in any areas examined. Changes to oxytocin and vasopressin receptors may underlie the behavioral changes observed and have translational implications for the mechanism of the increased risk of ASD subsequent to prenatal SSRI exposure.

## Introduction

In humans, antidepressant medication, most frequently a selective serotonin reuptake inhibitor (SSRI), is commonly prescribed to pregnant and lactating women with major depression (Boukhris et al., 2016). Use of SSRIs during pregnancy has increased dramatically over the last several decades, with estimates ranging from 6 to 13% of pregnancies in the United States (Cooper et al., 2007; Andrade et al., 2008; Alwan et al., 2011). Pharmacological treatment of maternal depression is typically recommended during the prenatal period, primarily because of the well-established negative effects of maternal depression (Davalos et al., 2012; Jarde et al., 2016). However, there may be side effects of SSRIs leading to preterm labor, altered gestational length and early delivery (Hayes et al., 2012), congenital heart malformations (Knudsen et al., 2014; Gentile, 2015a), persistent pulmonary hypertension (Grigoriadis et al., 2014), and adverse neurodevelopmental outcomes (El Marroun et al., 2014; Glover and Clinton, 2016). There is reason for concern about the effects of early exposure to SSRIs on the developing brain. SSRIs can cross the placental barrier (Hendrick et al., 2003; Rampono et al., 2009) and enter into breast milk (Kristensen et al., 1999; Rampono et al., 2000). Exposed infants show altered brain activity measured via EEG (Videman et al., 2017).

A growing body of research indicates increased rates of Autism Spectrum Disorder (ASD) in prenatally SSRI-exposed children (Croen et al., 2011; El Marroun et al., 2014; Gidaya et al., 2014; Gentile, 2015b; Boukhris et al., 2016; Andalib et al., 2017). While others have found no relationship when controlling for maternal factors (Hviid et al., 2013; Kobayashi et al., 2016) recent meta-analyses indicate that SSRI-exposure does increase autism diagnosis when pooling across studies (Man et al., 2015; Kaplan et al., 2017). Disentangling the effects of the underlying psychiatric condition of the mother from the effects of SSRIs on fetal development is difficult, and causality remains to be established.

Decades of research have indicated a link between ASD and serotonin, starting with the finding of hyperserotonemia in a subset of individuals shortly after the disorder was first described (Schain and Freedman, 1961). Hyperserotonemia has remained a consistent finding in a large subgroup of individuals diagnosed with ASD, with roughly one third of individuals presenting with high whole blood serotonin levels (Schain and Freedman, 1961; Anderson et al., 1987; Hranilovic et al., 2007; Gabriele et al., 2014; Muller et al., 2016). This finding has led researchers to suggest that hyperserotonemia underlies differences in the brain which are responsible for the appearance of autistic behavior (Whitaker-Azmitia, 2005; Yang et al., 2014). Animal models corroborate that hyperserotonemia leads to behavioral and neuroendocrine changes consistent with those seen in autism (Whitaker-Azmitia, 2005; McNamara et al., 2008; Veenstra-VanderWeele et al., 2012; Madden and Zup, 2014; Tanaka et al., 2018). Developmental hyperserotonemia decreases the number of oxytocinergic cells in the paraventricular nucleus of the hypothalamus in both rats (McNamara et al., 2008) and prairie voles (Martin et al., 2012), while decreasing affiliative behavior and increasing anxiety.

The effects of hyperserotonemia on the brain are rooted in serotonin’s critical role during early development as a trophic factor, long before it begins to function as a neurotransmitter. As a growth factor, it regulates development of its own and related systems and guides cell division, differentiation, migration, myelination, synaptogenesis, and dendritic pruning (Lauder, 1993; Azmitia, 2001; Wirth et al., 2017). Because serotonin exposure at this time also functions to autoregulate its own innervation throughout the brain via a negative feedback mechanism, developmental hyperserotonemia can cause organizational change which may enduringly alter serotonergic neurotransmission (Whitaker-Azmitia, 2001). Despite the relative paucity of serotonin neurons, they innervate almost all parts of the brain, making this system a powerful mediator of brain activity in many regions. Thus, alterations in serotonin during development may be particularly influential.

Significant overlap exists in psychiatric conditions associated with serotonin dysfunction and ASD. For instance, heightened rates of anxiety and depression may be seen in ASD populations (Lugnegård et al., 2011) and serotonin-based treatments, including SSRIs, show efficacy in treating some symptoms of ASD (Kolevzon et al., 2006; Hollander et al., 2012). Furthermore, depletion of tryptophan, the serotonin precursor, worsens repetitive behavior symptoms in ASD (McDougle et al., 1993, 1996). In addition, gastrointestinal problems are prevalent in ASD (Adams et al., 2011; Chaidez et al., 2014; McElhanon et al., 2014), and serotonin is highly involved in gut motility (Sikander et al., 2009). These comorbidities suggest that disrupted serotonin signaling may underlie the neurobiology of autism.

The serotonin system has important interactions with other systems in the brain. One such example is the interaction seen in the serotonin and oxytocin (OT) systems, both during development and in adulthood. Animal models indicate these systems are anatomically interconnected. Fibers from the dorsal and median raphe project to the paraventricular (PVN) and supraoptic (SON) nuclei of the hypothalamus, where oxytocin receptors (OTR) are distributed around them (Emiliano et al., 2007). Serotonin acts on OT neurons via serotonin receptors located in the PVN and SON, where OT is produced (Osei-Owusu et al., 2005). Likewise, OT acts via OTR on serotonin neurons in the raphe nuclei, where serotonin is produced, which may mediate the release of serotonin and have a role in the anxiolytic effects of OT (Yoshida et al., 2009). While evidence suggests that these two neurochemical systems may be working in tandem, it is not yet clear how early SSRI use may affect neural OT.

Vasopressin (AVP) is structurally and genetically similar to OT, and both play a central role in modulating the development of normal social behavior (Carter, 2014). Direct approaches to target the oxytocinergic and vasopressinergic systems are aimed at treating social dysfunction in disorders such as ASD. Although clinical results remain contradictory regarding whether effects are prosocial or antisocial (De Dreu et al., 2010; Guastella et al., 2010), recent advances in our understanding of the complex neurobiology of OT and AVP signaling, release, and degradation present promising avenues for understanding social function in ASD.

Animal models are useful in establishing causal links to long-term effects of perinatal SSRI exposure on social behavior in offspring (Zucker, 2017). Results are complicated by age, sex, and context-specific effects. Pre- and postnatal FLX exposure resulted in loss of a preference for a social partner vs. an empty cage, and a deficit in social recognition, in mice (Bond et al., 2020). When rats were tested as pre-adolescents, prior exposure to perinatal FLX prevented effects of maternal stress on play behavior in both sexes, but also resulted in an increase in aggressive play in males only (Gemmel et al., 2017). When tested as adults, perinatal exposure resulted in sex-specific increases in social behaviors (Gemmel et al., 2019). Another study of perinatal exposure found decreases in social interaction in male rats when tested as adults (Silva et al., 2018). In addition, some types of social behavior (i.e., pair bonding) are not present in rats and mice, necessitating a different animal model.

In the present study, we used the prairie vole as a translational model of developmental SSRI exposure. Prairie voles are socially monogamous microtine rodents that form lasting adult heterosexual pair bonds characterized by the formation of a partner preference, intrasexual aggression, and bi-parental care. Prairie voles are highly social and have a well described neurohypophyseal nonapeptide system (for review see Young et al., 2011) and can be tested in standardized assays of social behavior and anxiety-like behavior (e.g., partner preference, elevated plus maze). Here we use the prairie vole to examine how developmental exposure to a SSRI affects adult behavior and neural OTR, vasopressin 1a (V1aR), and serotonin 1A (5-HT1a) receptors and to determine if these changes replicate aspects of the symptomology of ASD.

## Materials and Methods

### Subjects

Subjects were laboratory-housed prairie voles (*Microtus ochrogaster*) from the breeding colony at the University of California, Davis. This colony was derived from a lineage of stock which was wild-caught near Champaign, IL. Animals were housed on a 14:10 light dark cycle with lights on at 0600. Food (Purina high-fiber rabbit chow) and water were available *ad libitum* in the home cage. Breeding pairs and offspring prior to weaning were housed in large polycarbonate cages (44 cm × 22 cm × 16 cm) and were given compressed cotton nestlets for bedding. Offspring were weaned on postnatal (PND) 20 and housed in small polycarbonate cages (27 cm × 16 cm × 16 cm) throughout testing with a same-sex sibling when available and a similarly aged non-sibling when not. All procedures were reviewed and approved by the Institutional Animal Care and Use Committee of the University of California, Davis.

### Drugs

Fluoxetine hydrochloride (Sigma-Aldrich, St. Louis, MO, United States) was dissolved in isotonic saline in a concentration of 1 mg/ml. It was then filtered into sterile solution and injected subcutaneously at the nape of the neck in a dose of 5 mg/kg. This dose was chosen based on the literature and the results of our own prior dose finding study. Both 5 and 10 mg/kg doses of FLX are commonly used in other rodent studies for perinatal administration (Gemmel et al., 2017, 2019; Grieb and Ragan, 2019). In the prairie vole dose-finding study, we examined the effect of 5 mg/kg FLX, 10 mg/kg FLX, or saline (SAL) vehicle on forced swim behavior and sucrose preference in socially isolated adult female prairie voles. At 5 mg/kg, females struggled significantly less (when compared to SAL, *t*_36_ = −2.92, *p* = 0.005), and spent approximately 40% less time immobile (although this was not statistically significant). In contrast, at 10 mg/kg struggle behavior did not differ from SAL, and time spent immobile trended toward an increase (when compared to saline, *t*_37_ = 1.64, *p* = 0.106). We therefore determined that 5 mg/kg was a more appropriate dose for the current study (data are available in Supplementary Figure S1).

### Design and Procedures

**FIGURE 1:**
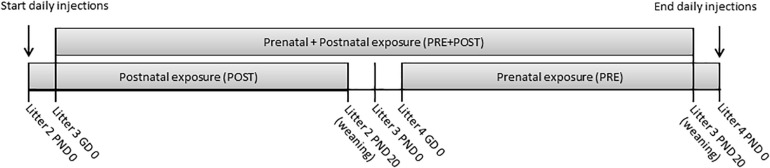
Timeline of maternal daily dosing and subject exposure. GD, gestational day; PND, postnatal day.

Virgin prairie voles (20 male, 20 female) were paired and allowed to raise a litter of pups together undisturbed. On the day of birth of the second litter, females were hand caught and pups were briefly removed. Litters were culled to two male and two female pups when possible. Females were given a subcutaneous injection of 5 mg/kg FLX or SAL at the nape of the neck and returned to the home cage along with her pups. On subsequent days, the female was hand caught and FLX or SAL was injected without removing the pups from the nipples. Females were dosed daily in this way with either FLX or SAL until the day of birth of the fourth litter. This design created three cohorts of FLX exposure: postnatal exposure in litter 2 (POST), both prenatal and postnatal exposure in litter 3 (PRE + POST), and prenatal exposure in litter 4 (PRE) (Figure 1). The average interbirth interval for litter 2–3 was 22.7 ±0.34 days (range 21–26), and for litter 3–4 was 22.9 ±0.19 days (range 21–24).

### Parental Care of Prenatally Exposed Offspring

Parental care is minimally altered following treatment with FLX (Villalba et al., 1997), however the effects of withdrawal prior to weaning has not been examined in prairie voles. Parental care of prenatally FLX-exposed subjects (litter 4) was quantified in the home cage to determine whether FLX withdrawal would significantly alter parental behavior. Undisturbed parental care was observed in the home cage for 20 min once during the morning and once in the afternoon on 2 days between PND 1-3. Behaviors were quantified in real-time using Behavior Tracker 1.5 (behaviortracker.com) using methods previously validated to measure the type and amount of parental care (Perkeybile et al., 2013). Both maternal and paternal behavior was measured, including huddling, non-huddling contact, licking/grooming, pup retrieval, nest building, and maternal nursing postures.

### Behavioral Tests

After weaning, subjects underwent behavioral testing. Half of each litter, one male and one female when possible, underwent behavioral testing during periadolescence, between PND21 and PND39. Periadolescent subjects underwent alloparental care, elevated plus maze, and open field testing in that order. The other half of each litter, one male and one female when possible, underwent behavioral testing as adults, between PND45 and PND120. Adult subjects were tested for alloparental care, elevated plus maze, and open field; in addition, they also underwent intrasexual adult affiliation and partner preference testing. All behaviors were quantified using Behavior Tracker 1.5 (behaviortracker.com). Behavioral tests occurred from 1 to 5 days apart.

### Alloparental Care

A minimum of 24 h after weaning, subjects were tested with a novel pup to measure alloparental care behavior as previously described (Bales et al., 2004a). Subjects were placed into an arena consisting of two polycarbonate cages (27 cm × 16 cm × 16 cm) connected by a short clear tube for a 45-minute acclimation period. This period was followed by a 10 min test in which a novel pup (PND 0-4) was placed into the arena. The subject was free to move about the arena and interact with the pup. Tests were video-recorded and later scored by a trained observer blind to condition. Behaviors quantified included frequency and latency of approach, sniffing, licking and grooming the pup, autogrooming, physical contact with the pup, huddling, pup retrievals, non-injurious biting, attacks, digging, and location in the arena relative to the pup. Digging and autogrooming were considered potential stereotypical behaviors. When attacks occurred, the test was immediately stopped and the subject removed from the arena. If possible, injuries were treated and the pup returned to the home cage. If necessary, the pup was euthanized. Each pup was used for no more than two test sessions. Following testing, animals were returned to their home cage.

Sex differences in prairie voles in this test are well-established, with males responding with higher levels of alloparental care than females. This sex difference, although already present in peri-adolescents, becomes more marked as animals become adult (Roberts et al., 1998).

### Elevated Plus Maze

The elevated plus-maze was used as a measure of anxiety and exploration (Insel et al., 1995) based on the rodent predisposition to prefer dark enclosed spaces (Campos et al., 2013). The maze consisted of two open and two enclosed opaque arms, each 67 cm long and 5.5 cm wide. The arms were elevated 1 m above the floor. Each vole was placed into the center of the maze and its behavior was scored for 5 min. Any animals that jumped off the open arms of the maze were captured and placed back into the center of the maze. If a subject jumped off the maze three times, the test was stopped. Throughout the course of the study, only four animals jumped off the maze, and data from only two animals had to be removed due to jumping. Trained observers blind to conditions scored behavior live for duration of time in the open and closed arms, freezing, and autogrooming with an inter-rater reliability greater than 90%. Autogrooming was considered a potential stereotypical behavior. Following testing animals were returned to their home cage.

It is worth noting that at baseline, prairie voles spend a higher amount of time in the open arms of the elevated plus-maze than mice typically do (Komada et al., 2008). While across 90 genetically engineered strains, mice spent an average of 9.19 ± 0.36% time in the open arms of the maze, prairie voles often spend 35–75% of their time in the open arms (Bales et al., 2004b; Greenberg et al., 2012). Male prairie voles tend to spend more time in the open arms, or exhibit higher frequencies of open arm entries, than females (Bales et al., 2004b; Greenberg et al., 2012).

### Open Field

The open field test was used as a second measure of anxiety and exploration (Ramos and Mormède, 1997). The open field consisted of a 40 cm × 40 cm × 40 cm plexiglass arena with a grid marked on the floor. The subject was placed in the center of the arena and behavior was digitally recorded for 10 min. Time spent in the center and the periphery was measured, as well as the frequency of rearing. Tests were video recorded and later scored using Behavior Tracker by trained observers with an inter-rater reliability greater than 90%. Following testing animals were returned to their home cage. Sex differences for prairie voles are not well established and are absent in some studies (Greenberg et al., 2012); we did not therefore predict any sex differences at baseline for this test.

### Intrasexual Adult Affiliation

Subjects were placed into a novel arena (27 cm × 16 cm × 16 cm) with a stimulus animal of the same sex and body size for 5 min as a low-threat, low-aggression social interaction task (Perkeybile and Bales, 2015). Behavior was video recorded and later scored by an observer blind to the treatment condition. The ethogram used to score behavior included affiliative behaviors (sniffing, physical contact, allogrooming, and play), anxiety related behaviors (rearing, digging, abrupt withdrawal), and aggressive behaviors (lunging, wrestling, chasing). Digging and autogrooming were considered potential stereotypical behaviors. Prior to testing, stimulus animals were screened for aggressive behavior with a novel animal, and were not used if they displayed high levels of aggression. Stimulus animals were collared prior to the start of testing to allow for identification during later behavioral scoring. Stimulus animals were used for a maximum of 2 tests, and were not reused if they experienced an aggressive interaction. Tests were continuously monitored for high levels of aggression and were stopped if necessary. Intense aggression was rarely seen. Following testing, animals were returned to the home cage. At baseline, we expected males to be more aggressive and less affiliative than females (Bales and Carter, 2003b).

### Partner Preference

This test is commonly used as an operational index of the formation of a pair-bond in the prairie vole (Williams et al., 1992; Bales and Carter, 2003a; Bales et al., 2013). Male subjects were housed with a female “partner” for 24 h prior to testing and female subjects were housed with a male partner for 6 h prior to testing. These durations have been previously shown to be sufficient time for the formation of a partner preference and account for the sex difference in time to pair bond formation (Williams et al., 1992; DeVries and Carter, 1999). Following this cohabitation, the opposite-sex mate of the subject (partner) and a non-related opposite-sex animal matched on age and weight to the mate (stranger) were tethered in opposing ends of a three-chamber testing apparatus. The subject was placed untethered in the empty middle chamber and was free to move about all three chambers and interact with either the partner or stranger for 3 h. The test was digitally recorded, and the duration of time in each of the three locations was quantified, as was the duration of side by side contact with the stranger and partner.

### Brain Extraction and Tissue Sectioning

Brains were taken from behaviorally tested animals of both ages (juvenile and adult), but only brains from the PRE + POST exposure cohort were analyzed for receptor binding (see below). Twenty-four hours after completion of all behavioral testing, subjects were euthanized via cervical dislocation and rapid decapitation under deep anesthesia. Brains were removed quickly and placed in powdered dry ice and then stored at −80°C until sectioning. Brain tissue was sectioned coronally in 20 μm slices at 20°C on a cryostat (Leica) and thaw mounted on Fisher Superfrost Plus slides. Slides were stored at −80°C until the time of assay.

### OTR and V1aR Autoradiography

Because they showed the largest effects on behavior, quantitative receptor autoradiography for OTR, V1aR, and 5-HT1aR was performed for the PRE + POST exposure cohort. Analyses were carried out on the right side of the brain only, as tissue punches were taken from the left side for additional analyses. Tissue was allowed to thaw in slide boxes containing desiccant packets. OTR and V1aR autoradiography was performed as previously reported (Perkeybile and Bales, 2015) with minor adjustments. For OTR binding, the ligand ^125^I-OVTA [^125^I-ornithine vasotocin [d(CH_2_)_5_[Tyr(Me)^2^, Thr^4^, Orn^8^, (^125^I)Tyr^9^-NH_2_] analog], 2200Ci/mmol (Perkin Elmer, Waltham, MA, United States) was used. For V1aR binding, the ligand ^125^I-LVA [^125^I-lin-vasopressin [^125^I-phenylacetyl-D-Tyr(ME)-Phe-Gln-Asn-Arg-Pro-Arg-Tyr-NH_2_] analog], 2200Ci/mmol (Perkin Elmer, Waltham, MA, United States) was used. After assay completion, slides along with ^125^I-autoradiographic standards (American Radiolabeled Chemicals, St. Louis, MO, United States) were exposed to Biomax MR film (Kodak, Rochester, NY, United States) for 72 h and then developed. We have previously reported a sex difference in the nucleus accumbens shell, with males displaying higher OTR binding than females at baseline (Guoynes et al., 2018).

### 5-HT_1A_ Autoradiography

For 5-HT_1A_ binding, 3.0 nM [^3^H]WAY-100635, 74Ci/mmol (Perkin Elmer, Waltham, MA, United States) was used. Tissue was rinsed in 50 mM Tris–HCl buffer (pH 7.5) followed by a 120 min incubation in the tracer buffer at room temperature. 10 nM of L-485,870, a dopamine antagonist, was included to prevent binding of WAY-100635 to Dopamine D4 receptors. Following the incubation period, tissue was rinsed twice in 50 mM Tris buffer at 4°C and then dipped in dH_2_O and air dried. Tissue was exposed to Carestream BioMax MR Film (Kodak, Rochester, NY, United States) for 6 weeks with ^3^H microscale standards (American Radiolabeled Chemicals, St. Louis, MO, United States). We had no *a priori* predictions as far as 5-HT_1A_ binding sex differences at baseline for this species.

### Quantification

Experimenters were blind to conditions during autoradiogram quantification. ImageJ software (National Institutes of Health, Bethesda, MD, United States) was used to quantify OTR optical binding density (OBD) in previously reported (Insel and Shapiro, 1992) regions of interest (ROI) including the nucleus accumbens core and shell, anterior central amygdala, and the lateral septum, and for V1aR in the medial amygdala, lateral septum, and ventral pallidum. 5-HT1aR OBD were quantified in the anterior and posterior lateral septum, dorsal hippocampus, dorsal raphe, and frontal cortex using MCID Core Digital Densitometry system (Cambridge, United Kingdom). The ten standard OBD values were used to generate a standard curve. Three separate measurements for ROIs and background OBD were averaged to yield normalized values and account for individual variation in background across samples.

### Data Analysis

Statistical analyses were conducted using SAS 9.4 (SAS Institute, Cary, NC, United States). All analyses were carried out using generalized linear mixed models (GLMM) utilizing backward selection to eliminate non-significant variables from the model. Significance level was set at *p* < 0.05 for all analyses and all tests were two-tailed. Data were checked for normality, and if not normally distributed, square root, quad root, or reciprocal transformation was used. If data was not transformable to normality, a GLMM was still used as recommended by Feir-Walsh and Toothaker (1974). *Post hoc* analyses utilized least squares means when the omnibus test was significant. The random factor used in all analyses was a pair ID (for the subject’s parents) to account for differences due to parenting or genetic background for subjects within the same litter or across litters. Drug condition was nested within this term, as each female maintained a consistent drug condition throughout the study and thus all offspring of a given pair had the same drug condition. When a three-way interaction was statistically significant, all two-way interactions which included the variables in the three-way interaction were left in the model even if not significant.

### Parental Care

A multivariate mixed model was used for analysis of parental care behavior. All three types of nursing were included in one model, as were behaviors that were examined concomitantly in both mothers and fathers that were not independent, such as huddling. Factors included in the model were pair ID and drug condition of the mother prior to cessation of treatment, as well as age of pups at observation and time of day as covariates.

### Alloparental Care Test

For the alloparental care analyses, variables were summed for duration of time in the same location (with the pup) or different location (without the pup) in the testing arena. A ratio was created to examine relative proportion of time spent in the same location as the pup relative to duration in a different location than the pup using the equation: ratio = with the pup/(with the pup + without the pup). Factors included in the model were pair ID, drug condition, sex, exposure cohort, age group, and interactions of these factors. Also analyzed were time spent in contact to the pup, time spent retrieving the pup, time spent in proximity to the pup, latency to approach, duration of social investigation, duration of licking, and duration of huddling over the pup.

### Elevated Plus Maze

For the elevated plus maze analysis, a ratio was created to examine the proportion of time spent on the open arms relative to total time on the maze using the equation: ratio = time on open arms/(time on open arms + time on closed arms). Factors included in the model were pair ID, drug condition, sex, exposure cohort, age group, and interactions of these factors. Autogrooming, entries onto the arms of the maze, and duration of freezing, were also analyzed.

### Open Field Test

For the open field test analyses, a ratio was created to examine proportion of time spent in the center of the arena relative to total time using the equation: ratio = time in center/(time in center + time in periphery). Factors included in the model were pair ID, drug condition, sex, exposure cohort, age group, and interactions of these factors. Rearing was also analyzed.

### Intrasexual Adult Affiliation

For the intrasexual adult affiliation analyses, the frequency of aggressive behavior was calculated by summing the frequencies of lunging and wrestling. Factors included in the model for each behavior (including affiliative, anxiety-like, and aggressive behaviors, as described above) were pair ID, drug condition, sex, exposure cohort, and interactions of these factors.

### Partner Preference Test

For between-group partner preference test analyses, a difference score was created to examine duration of time spent in the same cage as the partner relative to time spent with the stranger using the equation: difference = time with partner - time with stranger. The same procedure was used to examine physical contact with the partner relative to contact with the stranger using the equation: difference = time in contact with the partner - time in contact with the stranger. Duration of time spent in the empty chamber was analyzed separately, and square root transformed for analyses to make the residuals for this model normally distributed. Factors included in the model were pair ID, drug condition, sex, exposure cohort, and interactions of these factors.

Within-group partner preference analyses for the SAL and FLX groups were performed using matched t-tests for time spent in contact with the partner vs. time spent in contact with the stranger.

### Oxytocin, Vasopressin 1a, and Serotonin 1a Receptor Binding

For all binding analyses, density of binding in three sequential areas of each ROI were averaged for each individual. The model included pair ID, drug condition, sex, age group, and interactions of these factors.

Pearson correlations were calculated for the 4 ROIs quantified for OTR and the 3 ROIs quantified for V1aR with difference in time in physical contact and duration of time in the empty chamber in the partner preference test. Correlation of OTRs in the central amygdala and proportion of time on the open arms of the elevated plus maze was also examined. When multiple comparisons were made within a single behavioral or neuroanatomical test, a Benjamini-Hochberg false discovery rate adjustment for multiple comparisons was used (Benjamini and Hochberg, 1995).

## Results

### Parental Care

**FIGURE 2:**
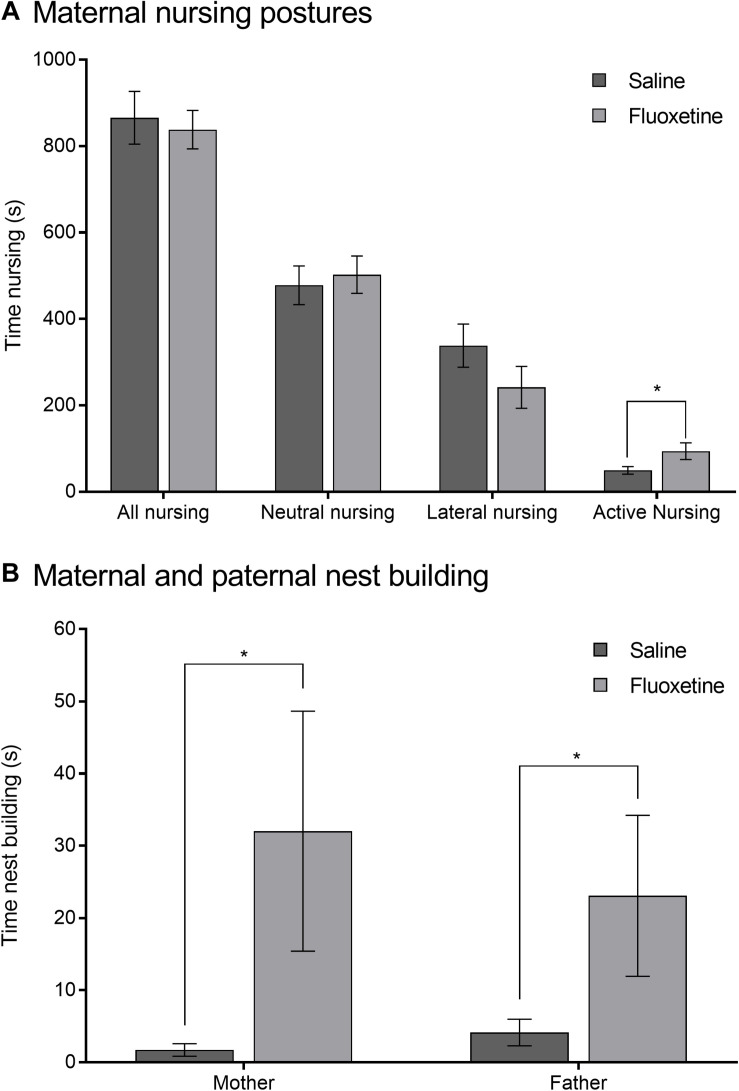
Parental care of prenatal exposure subjects. **(A)** Mean (±SEM) total, neutral, lateral, and active nursing duration comparing mothers previously exposed to saline to mothers previously exposed to fluoxetine. **(B)** Mean (±SEM) duration of nest building in mothers previously exposed to saline and their male pair-mates (fathers) compared to mothers previously exposed to fluoxetine and their pair-mates. *p < 0.05.

Parental care of the PRE cohort was minimally altered by the drug condition of the mother, either FLX withdrawal or no withdrawal from SAL at the time of parenting. Drug condition did not alter total duration of nursing, nor did it alter duration of neutral nursing postures or lateral nursing postures. However, duration of active nursing was altered by drug condition (*F*_1,51_ = 5.11, *p* < 0.05), with FLX-withdrawing dams spending more time in active nursing than those who had been treated with SAL (Figure 2A). Nest building duration was also greater in FLX-withdrawing mothers (*F*_1,51_ = 4.06, *p* < 0.05) as well as their untreated male pair-mates (*F*_1,51_ = 4.79, *p* < 0.05) compared to pairs in which mothers were previously treated with SAL (Figure 2B). Because of the high amount of variability in this behavior, we also analyzed nest-building with a non-parametric Kruskal-Wallis test. The duration of nest-building in FLX-withdrawing mothers, compared to SAL mothers, remained significant (χ^2^_1_ = 4.62, *p* < 0.05), however, the effect was non-significant in their male mates (χ^2^_1_ = 1.14, *p* > 0.05). All other behaviors observed were not affected by drug condition including maternal huddling, paternal huddling, maternal non-huddling contact, paternal non-huddling contact, maternal licking and grooming, paternal licking and grooming, maternal pup retrieval, paternal pup retrieval, maternal autogrooming, or paternal autogrooming.

### Behavior of Developmentally Exposed Offspring

#### Alloparental Care Test

**FIGURE 3:**
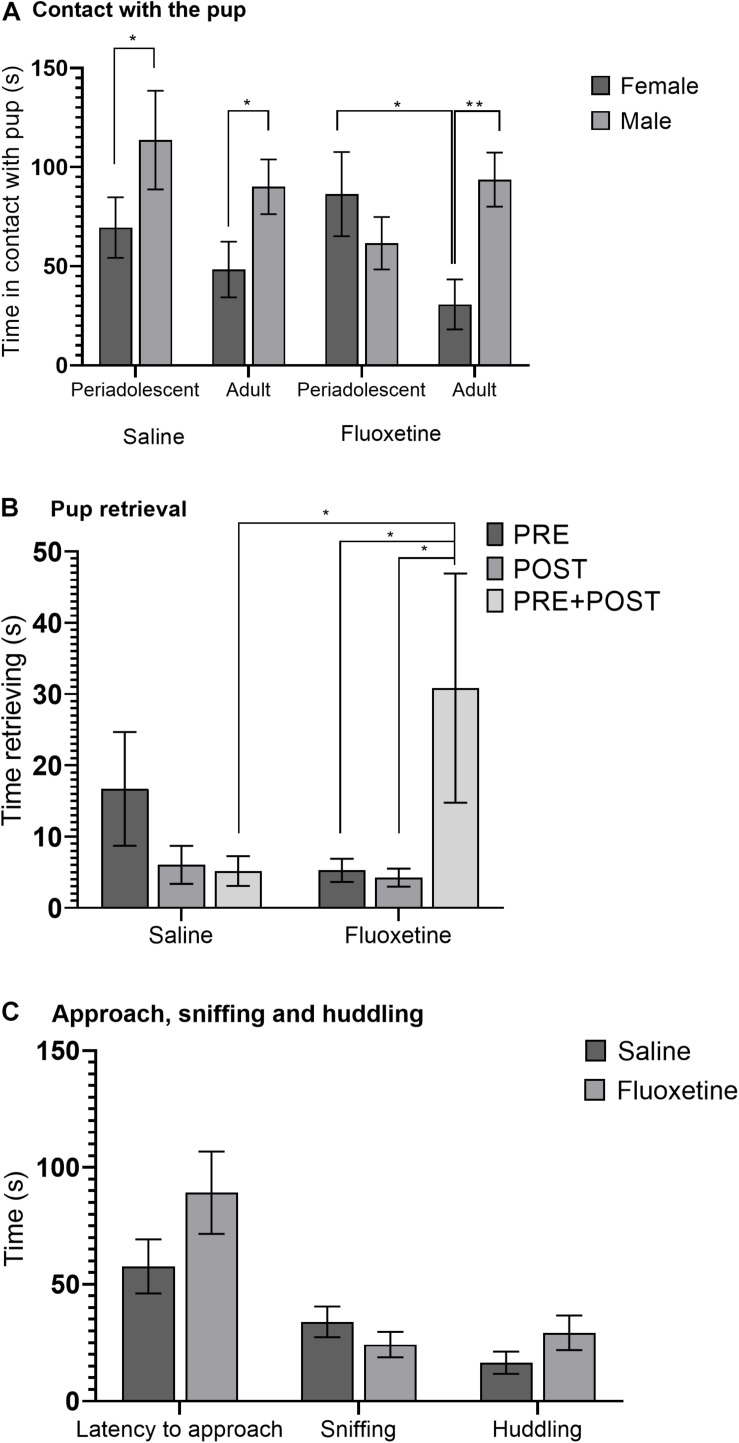
Alloparental care behavior. **(A)** Mean (± SEM) duration of physical contact with the pup comparing saline and fluoxetine exposure by age and sex. **(B)** Mean (±SEM) duration of pup retrieval comparing saline and fluoxetine exposure by exposure cohort. **(C)** Mean (± SEM) latency to approach the pup, sniffing, and huddling comparing saline and fluoxetine exposure. *p < 0.05, **p < 0.01.

Duration of overall pup physical contact was greater in males than in females (*F*_1,167_ = 8.28, *p* < 0.01). A three-way interaction of condition, sex, and age group (*F*_1,167_ = 3.77, *p* < 0.05) indicated that among FLX subjects, adult females were in contact with the pup less than periadolescent females (*t_41_* = 2.88, *p* < 0.05) and that among SAL subjects, periadolescent females were in contact with the pup less than periadolescent males (*t_49_* = 2.06, *p* < 0.05). Adult females spent less time in contact with the pup compared to adult males exposed to either SAL (*t_52_* = 1.97, *p* < 0.05) or FLX (*t_44_* = 2.83, *p* < 0.01) (Figure 3A). Put another way, females were in contact with the pup less than males under matching conditions, with the exception of FLX periadolescent females, which spent more time in contact with the pup than did FLX periadolescent males.

Duration of time spent retrieving the pup tended to be greater in males than in females (*F*_1,163_ = 3.69, *p* = 0.057). A drug condition by cohort interaction (*F*_2,163_ = 3.44, *p* < 0.05) (Figure 3B) indicated that in the PRE + POST cohort, FLX subjects spent more time retrieving the pup than SAL subjects (*t_63_* = 2.34, *p* < 0.05), and that in FLX subjects, PRE + POST subjects spent more time retrieving than PRE (*t_60_* = 2.40, *p* < 0.05) and POST (*t_58_* = 2.47, *p* < 0.05) subjects.

Fluoxetine exposure had no effect on proximity to the pup, licking the pup, latency of approach, social investigation, or huddling (Figure 3C). Ratio of time spent in the same chamber of the testing arena as the pup relative to total time was not altered by drug condition, nor was latency to approach the pup, duration of sniffing, huddling, licking, or grooming of the pup. There was no indication of heightened repetitive behavior with FLX exposure, and duration of autogrooming and digging were not altered by drug condition.

#### Elevated Plus Maze

**FIGURE 4:**
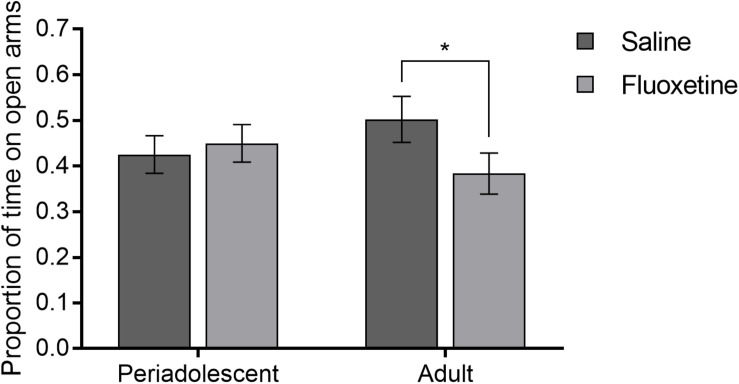
Elevated plus maze. Mean (±SEM) proportion of time spent in the open arms relative to total time comparing saline and fluoxetine exposure by age. *p < 0.05.

Proportion of time spent in the open arms relative to total time on the maze showed an interaction of drug condition and age group (*F*_1,141_ = 4.02, *p* < 0.05) such that FLX-exposed adults spent a lower proportion of time in the open arms compared to SAL-exposed adults (*t_64_* = 2.21, *p* < 0.05), while there was no such difference in periadolescent subjects (Figure 4). Drug condition did not alter the number of entries onto the arms of the maze, duration of freezing, or duration of autogrooming.

#### Open Field Test

**FIGURE 5:**
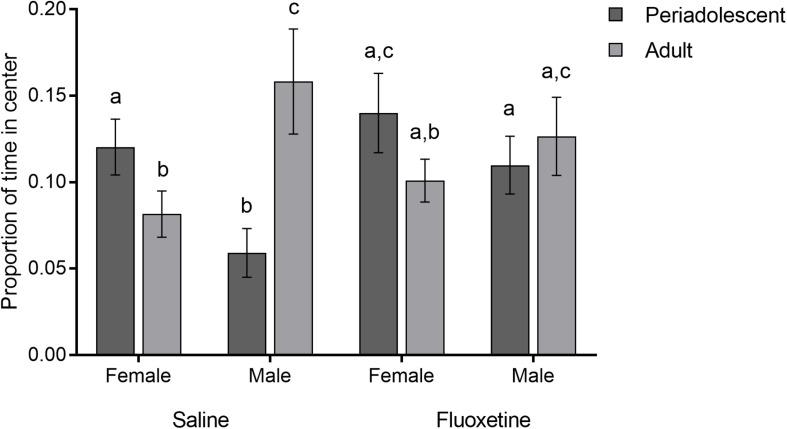
Open field test. Mean (±SEM) proportion of time spent in the center relative to total time comparing saline and fluoxetine exposure by age and sex. Different letters indicate a significant difference at *p* < 0.05.

Proportion of time spent in the center of the open field relative to total time showed a three-way interaction of drug condition, sex, and age group (*F*_4,119_ = 4.66, *p* < 0.01) (Figure 5). In SAL-exposed females, periadolescents spent more time in the center than adults (*t_39_* = 2.48, *p* = 0.01), while this was not true for FLX-exposed subjects (*t_30_* = 1.29, *p* = 0.20). Among SAL exposed subjects, time in the center was greater in adult males than adult females (*t_31_* = 3.42, *p* < 0.001), in periadolescent females than periadolescent males (*t_44_* = 1.94, *p* = 0.05), and in adult males than periadolescent males (*t_36_* = 3.00, *p* < 0.01). There was also a trend level difference between SAL males and SAL females (*t_76_* = 1.91, *p* = 0.06). There were no sex or age group differences within the FLX-exposed subjects. Duration of autogrooming and frequency of rearing were not affected by drug condition.

#### Intrasexual Adult Affiliation Test

Duration of sniffing of the stimulus animal, the primary form of social investigation, did not differ by drug condition. Duration of allogrooming of the stimulus animal showed a trend level interaction of drug condition and sex (*F*_1,91_ = 3.73, *p* = 0.057). FLX exposed males spent more time allogrooming than SAL exposed males (*t_49_* = 1.77, *p* = 0.07), and SAL females spent more time allogrooming than SAL males (*t_48_* = 1.91, *p* = 0.059). Duration of time in physical contact with the stimulus animal, autogrooming, or frequency of rearing were not altered by drug condition.

**FIGURE 6:**
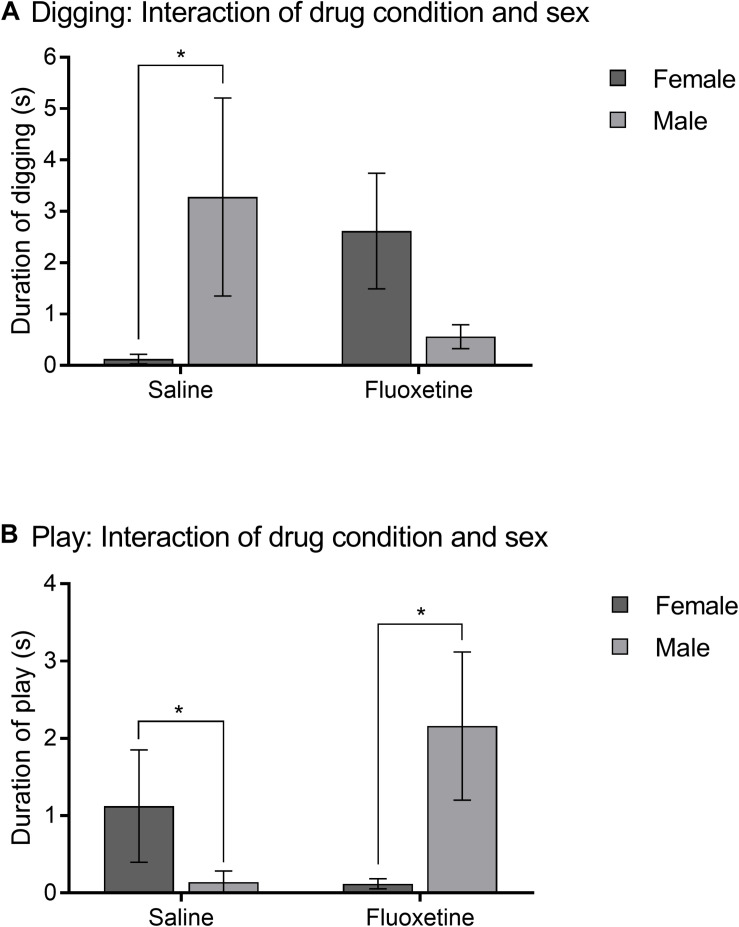
Intrasexual adult affiliation. **(A)** Mean (± SEM) duration of digging comparing saline and fluoxetine exposure by sex. **(B)** Mean (±SEM) duration of play comparing saline and fluoxetine exposure by sex. *p < 0.05.

Frequency of aggressive behavior was not altered by drug condition. In contrast, duration of digging showed an interaction of treatment and sex (*F*_1,73_ = 4.62, *p* < 0.05) (Figure 6A). SAL males dug more than SAL females (*t_48_* = 2.53, *p* < 0.05), but there was no sex difference in FLX exposed subjects.

Duration of play with the stimulus animal showed an interaction of drug condition and sex (*F*_1,91_ = 5.75, *p* < 0.05) (Figure 6B). FLX males played more than FLX females (*t_45_* = 2.23, *p* < 0.05) and SAL males (*t_49_* = 2.36, *p* < 0.05).

### Partner Preference Test

**FIGURE 7:**
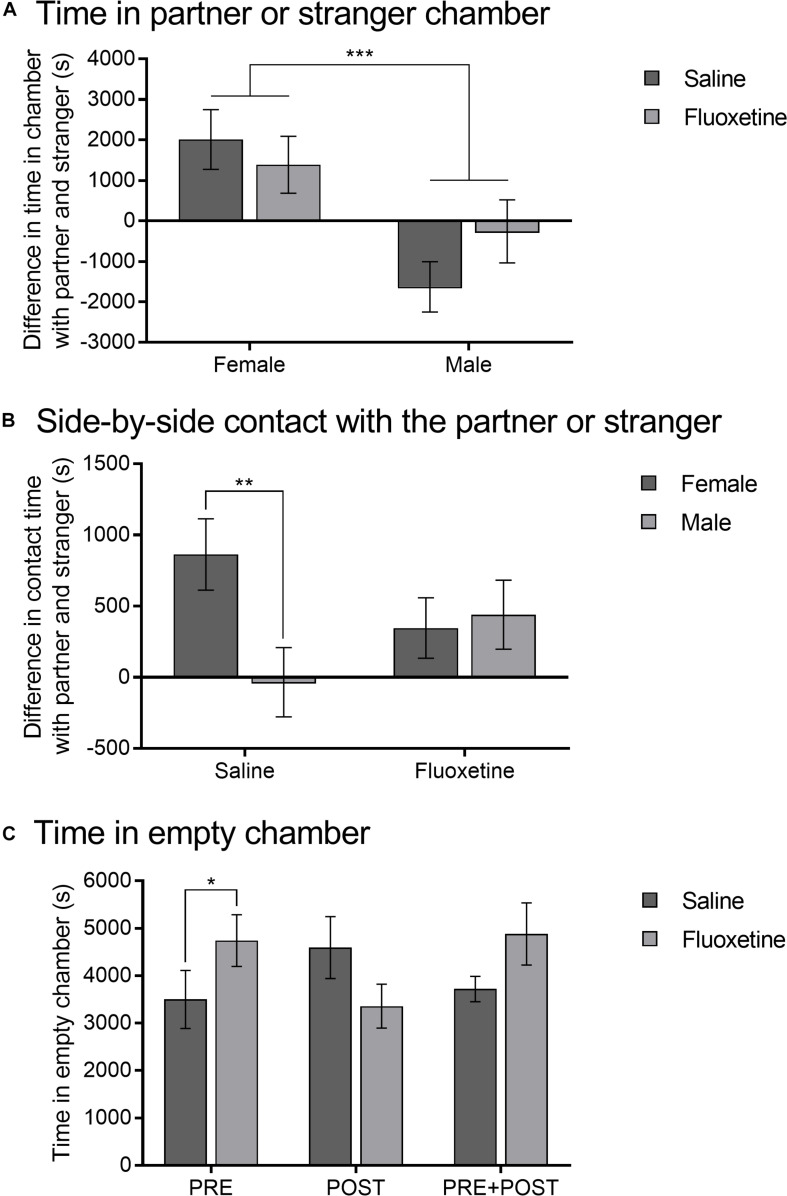
Partner preference test. **(A)** Mean (±SEM) difference in duration between time spent in the partner chamber and the stranger chamber comparing saline and fluoxetine exposure by sex. **(B)** Mean (±SEM) difference in duration between time spent in side-by-side contact with the pair-mate and the stranger comparing saline and fluoxetine exposure by sex. **(C)** Mean (±SEM) duration of time in the empty chamber comparing saline and fluoxetine exposure by exposure cohort. *p < 0.05, **p < 0.01, ***p < 0.001.

Difference in duration of time in the partner and stranger chambers was greater in females compared to males (*F*_1,74_ = 12.95, *p* < 0.001) but did not differ by cohort or drug condition (Figure 7A). Difference in duration of time in side-by-side contact with the partner and the stranger was not altered by cohort but did show an interaction of sex and drug condition (*F*_1,73_ = 4.01, *p* < 0.05) (Figure 7B). SAL females spent more time in physical contact with the partner than SAL males (*t_40_* = 2.62, *p* < 0.01), but there was no sex difference in the FLX condition. Within the SAL group, females formed a significant preference for the partner (*t_24_* = 3.44, *p* = 0.002), while males did not (*t*_16_ = −0.14, *p* = 0.891). Within the FLX group, neither females (*t*_18_ = 1.672, *p* = 0.121) nor males (*t*_16_ = 1.816, *p* = 0.07) formed a significant preference.

Duration of time spent in the empty chamber in the partner preference test showed an interaction of drug condition and exposure cohort (*F*_2,70_ = 4.17, *p* < 0.05) (Figure 7C). Subjects in the PRE cohort that were exposed to FLX spent more time in the empty chamber than those exposed to SAL (*t_26_* = 2.06, *p* < 0.05). Time in the empty chamber was not altered by sex, nor were there differences by drug condition in the PRE + POST or POST conditions.

### Quantitative Receptor Autoradiography

#### Oxytocin Receptors

**FIGURE 8:**
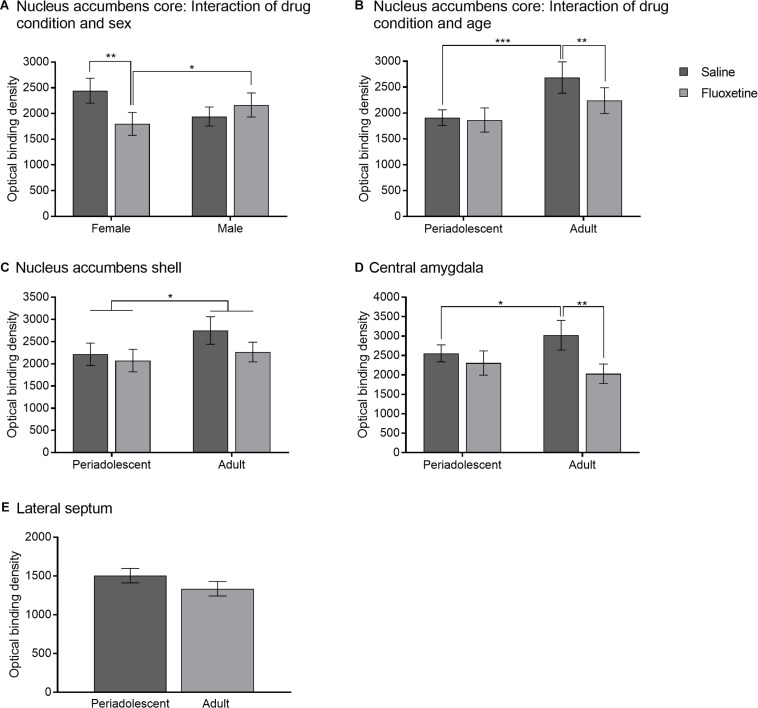
Oxytocin receptor binding. **(A)** Mean (± SEM) optical binding density in the nucleus accumbens core comparing saline and fluoxetine exposure by sex. **(B)** Mean (±SEM) optical binding density in the nucleus accumbens core comparing saline and fluoxetine exposure by age. **(C)** Mean (±SEM) optical binding density in the nucleus accumbens shell comparing saline and fluoxetine exposure by age. **(D)** Mean (±SEM) optical binding density in the central amygdala comparing saline and fluoxetine exposure by age. **(E)** Mean (±SEM) optical binding density in the lateral septum comparing saline and fluoxetine exposure. *p < 0.05, **p < 0.01, ***p < 0.001.

**FIGURE 9:**
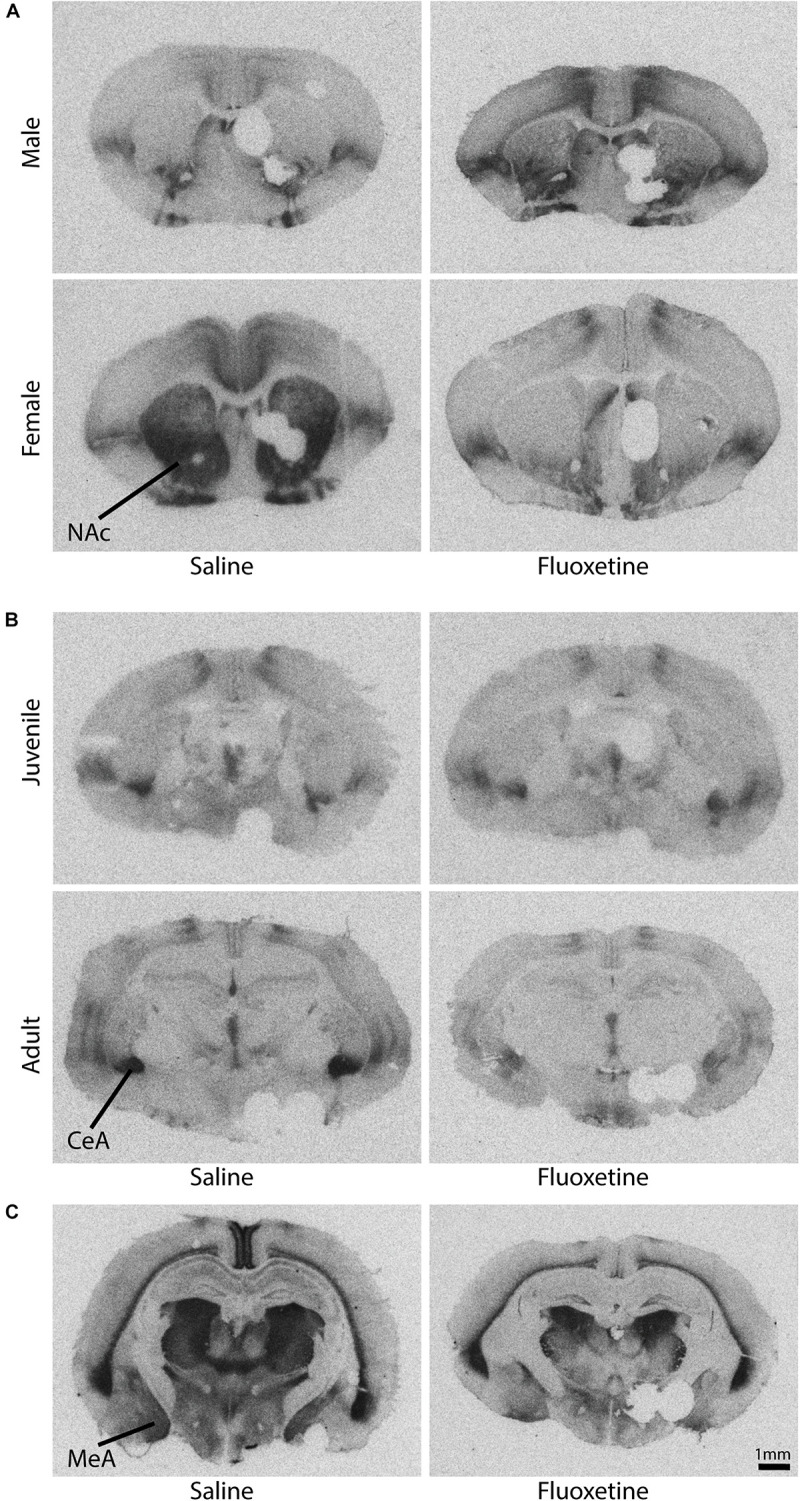
Representative autoradiograms of oxytocin and vasopressin 1a receptor binding. Please note that tissue punches were taken from the left side of each brain to assess additional outcome measures not reported here. **(A)** Oxytocin receptor binding in the nucleus accumbens core shows a sex by drug condition interaction (see also Figure 8A). **(B)** Oxytocin receptor binding in the central amygdala shows an age by drug condition interaction (see also Figure 8C). **(C)** Vasopressin 1a receptor binding in the medial amygdala shows a drug condition effect (see also Figure 9A).

Oxytocin receptors binding in the nucleus accumbens core was lower in FLX subjects compared to SAL subjects (*F*_1,43_ = 3.96, *p* = 0.05) and was greater in adult compared to periadolescent subjects (*F*_1,43_ = 7.18, *p* < 0.01). A drug condition by sex interaction (*F*_1,43_ = 4.89, *p* < 0.05) (Figures 8A, 9A) indicated that FLX females had less OTR binding than SAL females (*t_31_* = 2.84, *p* < 0.01) and FLX males (*t_30_* = 2.20, *p* < 0.05). A drug condition by age group interaction (*F*_1,43_ = 5.02, *p* < 0.05) (Figure 8B) indicated that FLX adults had less OTR binding than SAL adults (*t_28_* = 2.73, *p* < 0.01). Adults also had greater OTR binding compared to periadolescents with SAL exposure (*t_34_* = 3.50, *p* = 0.001), but this was not the case with FLX exposure (*t_30_* = 0.31, *p* = 0.76). OTR binding in the nucleus accumbens shell did not differ by drug condition or sex. Adult subjects had greater OTR binding in the nucleus accumbens shell than periadolescents (*F*_1,45_ = 3.92, *p* = 0.05; Figure 8C).

Oxytocin receptors binding in the anterior central amygdala was decreased with FLX exposure compared to SAL exposure (*F*_1,46_ = 8.42, *p* < 0.01). There was no effect of sex on OTR binding in the central amygdala. A condition by age group interaction (*F*_1,46_ = 3.98, *p* = 0.05) (Figures 8D, 9B) indicated that FLX adults had lower OTR binding compared to SAL adults (*t_66_* = 3.26, *p* < 0.01), and that SAL adults had higher OTR binding than SAL periadolescents (*t_34_* = 2.01, *p* = 0.05), but this age difference was not found with FLX exposure. OTR binding in the lateral septum was not altered by drug condition (Figure 8E), sex, or age group.

Oxytocin receptors binding did not correlate with difference in contact between the partner and stranger or duration in the empty chamber in the partner preference test. There was also no correlation between OTR binding in the central amygdala and proportion of time on the open arms of the elevated plus maze.

#### Vasopressin 1a Receptors

**FIGURE 10:**
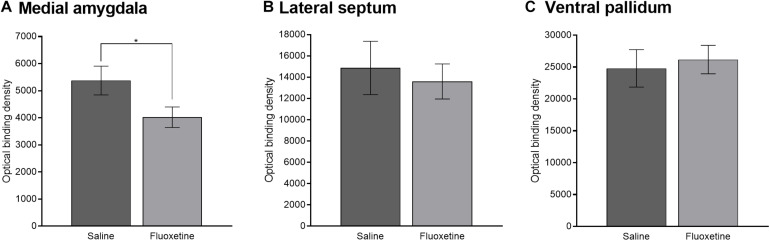
Vasopressin 1a receptor binding. **(A)** Mean (±SEM) optical binding density in the medial amygdala comparing saline and fluoxetine exposure. **(B)** Mean (±SEM) optical binding density in the lateral septum comparing saline and fluoxetine exposure. **(C)** Mean (±SEM) optical binding density in the ventral pallidum comparing saline and fluoxetine exposure. *p < 0.05.

Vasopressin 1a binding in the medial amygdala was reduced by FLX exposure compared to SAL exposure (*F*_1,47_ = 4.20, *p* < 0.05) (Figures 10A, 9C). V1aR binding in the medial amygdala was not altered by sex or age group. V1aR binding in the lateral septum was not altered by drug condition, sex, or age group (Figure 10B). V1aR binding in the ventral pallidum was not altered by drug condition, sex, or age group (Figure 10C).

Vasopressin 1a binding density in the three ROIs quantified did not correlate with difference in contact between the partner and stranger or duration in the empty chamber in the partner preference test once adjusted to account for multiple comparisons.

#### Serotonin 5-HT1a Receptors

**FIGURE 11:**
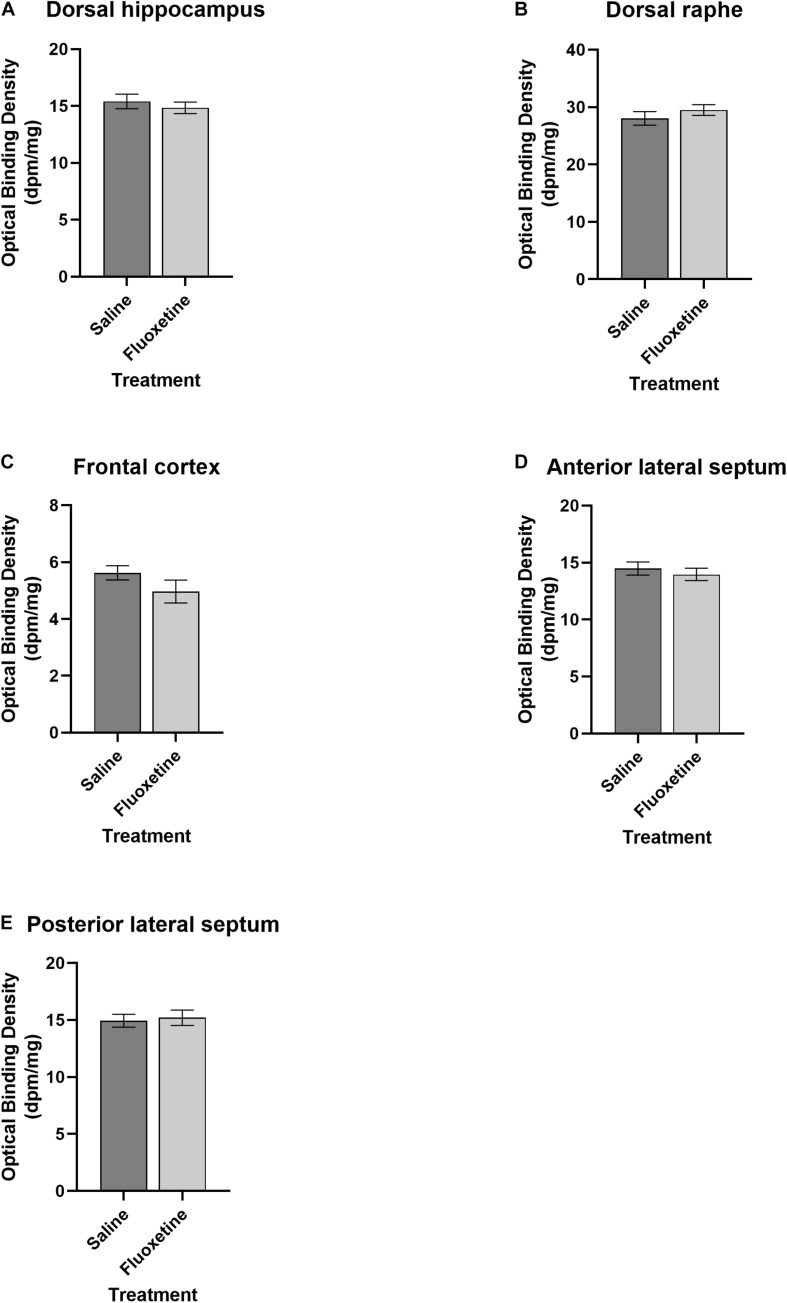
Serotonin receptor 1a binding. **(A)** Mean (±SEM) optical binding density in the dorsal hippocampus comparing saline and fluoxetine exposure. **(B)** Mean (±SEM) optical binding density in the dorsal raphe comparing saline and fluoxetine exposure. **(C)** Mean (±SEM) optical binding density in the frontal cortex comparing saline and fluoxetine exposure. **(D)** Mean (±SEM) optical binding density in the anterior lateral septum comparing saline and fluoxetine exposure. **(E)** Mean (±SEM) optical binding density in the posterior lateral septum comparing saline and fluoxetine exposure.

Unexpectedly, there was no effect of FLX exposure on 5-HT_1A_ receptor binding density in any ROI examined (anterior and posterior lateral septum, dorsal hippocampus, dorsal raphe, frontal cortex) nor were there any significant interactions of age group, sex, and ROI (Figures 11A–E).

## Discussion

Understanding the etiology of the increased risk of ASD associated with developmental SSRI exposure is an area of research which can greatly benefit from animal models. Here, we used the prairie vole as a translational model in which to examine how exposure to an SSRI, FLX, affects behavior, neuropeptide receptors, and serotonin receptors in the brain.

We examined three primary behavioral domains which are associated with ASD: social behavior, repetitive behavior, and anxiety-like behavior. The first two represent the two primary diagnostic criteria for ASD, impaired social communication and stereotyped or repetitive behavior; the third represents the heightened anxiety frequently comorbid in ASD (White et al., 2009; van Steensel et al., 2011). Modeling the social communication domain of ASD is particularly difficult in animal models. Verbal language is uniquely human, and thus the precise deficits found in individuals with ASD cannot be modeled in any animal species.

We examined sociality by measuring species-typical behaviors involved in social interaction and looking for deficits in FLX exposed subjects. Social investigation (sniffing) was not altered by FLX with a novel social partner, be it a pup or an adult conspecific. Affiliative behavior, which is ubiquitous in prairie voles, was altered by FLX exposure (Table 1). We observed changes in alloparental care (Figures 3A,B), in play behavior with a same-sex adult (Figure 6B), and in time spent in the empty chamber of the partner preference test (Figure 7C). The changes in alloparental care were primarily in retrieval behavior, with males that had been treated with both prenatal and postnatal FLX spending significantly more time retrieving (Figure 3B). These males were picking up the pup in their mouths and running excitedly around the test arena, in an apparently less organized manner of providing care for the pup.

**TABLE 1:** Summary of behavioral effects of fluoxetine exposure.

Behavioral test	Measure	Effect of fluoxetine	Interacts with	Results
Alloparental Care	Physical contact	Y	Sex, age group	FLX adult female < FLX peri female SAL peri female < SAL peri male
	Pup retrieval	Y	Exposure cohort	FLX PRE + POST > SAL PRE + POST FLX PRE + POST > FLX PRE, FLX POST
	Same chamber as pup	N	–	–
	Latency to approach	N	–	–
	Sniff	N	–	–
	Huddle	N	–	–
	Lick and groom	N	–	–
	Autogroom	N	–	–
	Dig	N	–	–
Elevated plus maze	Ratio of time on open arms	Y	Age	FLX adult < SAL adults
	Arm entries	N	–	–
	Freeze	N	–	–
	Autogroom	N	–	–
Open field test	Ratio of time in center	Y	Sex, age group	Eliminated sex and age differences seen in SAL
	Autogroom	N	–	–
	Rear	N	–	–
Intrasexual adult affiliation	Sniff	N	–	–
	Allogroom	Y	Sex	FLX male > SAL male (trend) Eliminated sex difference seen in SAL
	Physical contact	N	–	–
	Autogroom	N	–	–
	Rear	N	–	–
	Aggression	N	–	–
	Dig	Y	Sex	Eliminated sex difference seen in SAL
	Play	Y	Sex	FLX male > FLX female FLX male > SAL male
Partner preference test	Difference in partner and stranger chamber time	N	–	–
	Difference in side-by-side contact	Y	Sex	Eliminated sex difference seen in SAL
	Empty chamber time	Y	Exposure cohort	FLX PRE > PRE SAL

*Y, significant effect; N, no effect; peri, periadolescent.*

During the partner preference test, prenatal FLX exposure also led subjects of both sexes to opt out of social interaction in favor of time alone in the empty cage (Figure 7C), indicating that FLX led to a rejection of social interaction very atypical of prairie voles. However, FLX males also spent more time in play behavior with stimulus males during the intrasexual affiliation test. Much as the research in humans suggests, prenatal SSRI exposure may increase the likelihood of asociality, or the alteration or disorganization of sociality; but it does so in subtle, non-deterministic ways.

The neurohypophyseal nonapeptides, oxytocin and vasopressin, are likely candidates to be involved in such shifts in sociality due to their developmental interaction with serotonin as well as their important roles in social behavior across species (Carter and Perkeybile, 2018). We found that FLX exposure reduced the binding density of oxytocin receptors in the nucleus accumbens core and the central amygdala (Figures 8A,B,D), and the binding density of vasopressin 1a receptors in the medial amygdala (Figure 9A). While the nucleus accumbens shell has been strongly implicated in studies of prairie vole pair bonding, oxytocin receptors in the core are under-studied in the neurobiology of social behavior in voles, and may represent a new avenue of investigation.

It is likely that changes in OTR and AVPR1a underlie the differences found not only in social behavior, as described above, but also in anxiety-like behavior. Anxiety-like behavior was altered in the elevated plus maze (Figure 4), where adults spent less time on the open arms if developmentally exposed to FLX, regardless of the timing of exposure. This result is in line with previous research which has reported an increase in anxiety-like behavior in adults exposed to an SSRI developmentally (Ansorge et al., 2004; Boulle et al., 2016). We also found that FLX exposed subjects had lower OTR in the central amygdala during adulthood but not during periadolescence (Figure 8D). The amygdala is an area of the brain that is highly involved in anxiety and emotion regulation (Babaev et al., 2018). OTRs in the central amygdala are known to be involved in anxiety, as well as regulation of the hypothalamic-pituitary-adrenal axis, and can play a role in mediating the stress response (Neumann et al., 2000). Likewise, V1aR in the amygdala mediate stress and anxiety, with binding at V1aRs linked to heightened anxiety, reducing time spent in the open arms of the elevated plus maze (Hernández et al., 2016). Taken together, one potential mechanism by which developmental exposure to FLX increases anxiety in adulthood may be the reduction of OTRs and V1aRs in the amygdala.

While developmental FLX altered social and anxiety related behaviors, there was no indication of increased repetitive behaviors in FLX exposed subjects. We found no increase in stereotypies in any of the behavioral tests examined. Autogrooming and digging were not increased by FLX exposure in any of the behavioral tests in which they were measured.

Changes in offspring behavior may have been mediated by changes in the behavior of the mothers treated with FLX, although these were relatively subtle. In particular, mothers that were withdrawing from FLX spent extra time in active nursing (Figure 2A) and in nest-building (Figure 2B). The male pair mates of the FLX-withdrawing mothers also spent higher amounts of time in nest-building (although this effect was eliminated when the data were examined non-parametrically). Unfortunately, we missed the opportunity to assess the quality of the nests being produced (Figure 2B). Nest quality is an often-used measure of parental behavior in rodents and other species (Mann, 1993; Deacon, 2012). In three-spined sticklebacks, FLX reduced measures of male nest quality (Sebire et al., 2015); while in mice, females prenatally treated with FLX displayed lower nest quality during early days postpartum (Svirsky et al., 2016). The quality of the nest could affect various measures for the offspring including survival (Hamilton et al., 1997), thermoregulation (Gaskill et al., 2013), and even sleep (Harding et al., 2019). It is possible that the FLX-withdrawing parents put in extra time nest-building, while still producing low quality nests. A disorganized approach to nest-building would be consistent with the active nursing behavior of the mothers, which is when they locomote around the cage with the pups still attached to the nipples (prairie vole pups have milk teeth). Given that the pups are being bounced against substrate as they are dragged around, we have generally regarded this as a lower quality form of maternal behavior. Active nursing is also higher in prairie vole mothers that are broadly characterized as “low contact” mothers (Perkeybile et al., 2013). Future research on this topic should include nest quality as a variable in aiding understanding of the effects of FLX on parental behavior.

A major limitation of this study is that we did not find a partner preference in the SAL-treated males (Figure 7B). A possible explanation for this is that the daily injections inadvertently created a prenatal stress paradigm to which all subjects were exposed. Daily saline injections in pregnant rats have been shown to be sufficient to change several aspects of stress reactivity and the serotonin system in offspring (Peters, 1982). Prenatal stress has been shown to alter the social behavior of offspring (Weinstock, 2001; Schulz et al., 2011; Wilson and Terry, 2013) and likely prevented any of our animals from forming a preference. However, the finding that prenatally FLX exposed subjects spent more of their time alone compared to SAL treated animals suggests a change in social interest above and beyond that involved in the formation of a partner preference. Furthermore, maternal stress adds ecological validity given that in human prenatal SSRI use there is an underlying psychiatric condition for which pharmacological treatment with SSRIs has been prescribed. Chronic stress is frequently used in the laboratory to induce a learned helplessness phenotype of depressive-like behavior to model depression (Pollak et al., 2010).

An interesting and unexpected finding was that FLX exposure eliminated sex differences across multiple behavioral tests. One example is the change in physical contact with the pup seen in the alloparental care test (Figure 3A). Male prairie voles are typically more alloparental than females, and here we saw that with FLX exposure, male periadolescents were not more alloparental than females, as was the case with SAL exposure. Male alloparental care is directly impacted by estrogen receptor expression, and sex-dependent changes in alloparental care with increasing age are based on changes in estrogen receptor expression (Perry et al., 2015). FLX exposure also eliminated the sex difference in partner and stranger contact in the partner preference test (Figure 7B). Both alloparental care and partner preference are examples of behaviors that show well-established sex differences in prairie voles. Estrogen receptor α expression has been implicated in reducing heterosexual adult contact in the partner preference test as well as male alloparental care behavior (Lei et al., 2010). FLX has estrogenic effects both *in vivo* and *in vitro* (Jacobsen et al., 2015; Pop et al., 2015; Muller et al., 2016), as does its bioactive metabolite norfluoxetine (Lupu et al., 2015). There is evidence in the literature for sex-specific effects of FLX on estrogen receptor expression (Adzic et al., 2017). FLX may have altered estrogen receptor expression, which in turn reduced affiliative behavior specifically in males, thus abolishing the sex differences seen in the SAL exposure groups. Future work should more thoroughly characterize the effects of developmental FLX on steroid receptors to further understand its behavioral effects.

Developmental timing is likely to be important in SSRI exposure. While some work has suggested that in humans, any chronic exposure in the year prior to birth results in heightened risk (Croen et al., 2011), others have found that either the first or third trimester are the periods of greatest risk (Oberlander et al., 2008; Croen et al., 2011; Harrington et al., 2014). In order to address the effects of exposure timing, we evaluated behavior in three different gross exposure cohorts spanning prenatal and postnatal development. We found few effects of FLX that were specific to an exposure cohort with the notable exception of increased duration in the empty chamber of the partner preference test in the PRE cohort. It is likely that creating shorter dosing periods which translate to specific trimesters in human pregnancy would be beneficial to more accurately determining how to best limit risk to offspring based on timing of exposure.

It is also worth pointing out that due to study design, offspring with different exposure timing were born to mothers of different parity and were potentially subject to different maternal hormone exposures. For example, pups that were part of the PRE + POST cohort were being nursed by mothers which were becoming pregnant again. To the extent that variation in maternal hormones due to parity or pregnancy may have affected hormones during the postpartum estrus or lactation (Bridges and Byrnes, 2006; Bridges, 2016), altering pup hormonal exposure *in utero* or through milk, these exposures may have varied in this study. In addition, all subjects in that cohort were litter 3 for their parents, whereas subjects in the POST cohort were all litter 2, and subjects in the PRE cohort were all litter 4; which could have also had effects on hormone exposure.

We have shown here that developmental SSRI exposure alters OTR and AVPR1a, but not 5-HT_1A_, binding. Because FLX’s mechanism works to increase serotonin neurotransmission by blocking reuptake of serotonin, it was surprising to find that 5-HT_1A_ receptor binding was unchanged by FLX in all regions examined. Studies in mice have shown that perinatal FLX can regularize 5-HT_1A_ levels that have been altered by other developmental factors (Nagano et al., 2012; Stagni et al., 2015). For the current study, it appears that the behavioral effects were mediated by OTR and V1aR without concomitant changes in the 5HT system. However, while there was no change in serotonin receptor density, actions on OTR and V1aR subsequent to FLX exposure may have been precipitated by changes in the peptides themselves, the function or location of the receptor, or other downstream cellular mechanistic pathways. Serotonin developmentally autoregulates its own innervation throughout the brain (Herlenius and Lagercrantz, 2004) and is plastic throughout development. Fetal exposure to FLX is poorly understood, yet it is clear that it leads to changes that last well into adulthood (Kiryanova et al., 2013). While SSRIs are presumed to increase extracellular serotonin in the long term, short term SSRI exposure can reduce raphe cell firing by acting on autoreceptors leading to a reduction in extracellular serotonin (Tao et al., 2000). Such activity may have neurodevelopmental consequences for offspring that have yet to be elucidated fully, but which warrant further investigation.

The serotonin system is also an extensive system with 15 different types of receptors (Carr and Lucki, 2011). We chose to examine the 1A receptor because of its autoreceptor function, but it may be the case that other exclusively post-synaptic serotonin receptors were altered while 1A was not. Further work examining other serotonin receptor populations will be important to clarify how serotonergic neurotransmission is altered by SSRI use prenatally. It is also possible that species differences between mice and voles may have altered the effects of FLX on 5-HT_1A_ receptor binding.

Another area that should be considered is how exposure interacts with the maternal and early postnatal environment, as environmental moderation of SSRI effects may underlie their effects (Alboni et al., 2017). Since the prevalent and incident use of SSRI-exposed pregnancies has increased in the last two decades (Alwan et al., 2011), it is of the utmost importance that we more clearly understand the causes and consequences that prenatal SSRI exposure may have on the developing brain.

## Data Availability Statement

The raw data supporting the conclusions of this article will be made available by the authors, without undue reservation.

## Ethics Statement

The animal study was reviewed and approved by Institutional Animal Care and Use Committee of the University of California, Davis.

## Author Contributions

RL and KB designed the research. RL, MP, CG, and SF conducted the experiments. RL, SF, and KB analyzed the data. RL wrote the first draft of the manuscript. All authors edited the manuscript.

## Conflict of Interest

The reviewer CH declared a shared affiliation, with no collaboration, with one of the authors, MP, to the handling editor at the time of review. The remaining authors declare that the research was conducted in the absence of any commercial or financial relationships that could be construed as a potential conflict of interest.
