# Supplementary figures and images for: Developmental Fluoxetine Exposure Alters Behavior and Neuropeptide Receptors in the Prairie Vole
Source: Front Behav Neurosci. 2020 Nov 16;14:584731. doi: 10.3389/fnbeh.2020.584731 (PMC7701284; doi:10.3389/fnbeh.2020.584731)

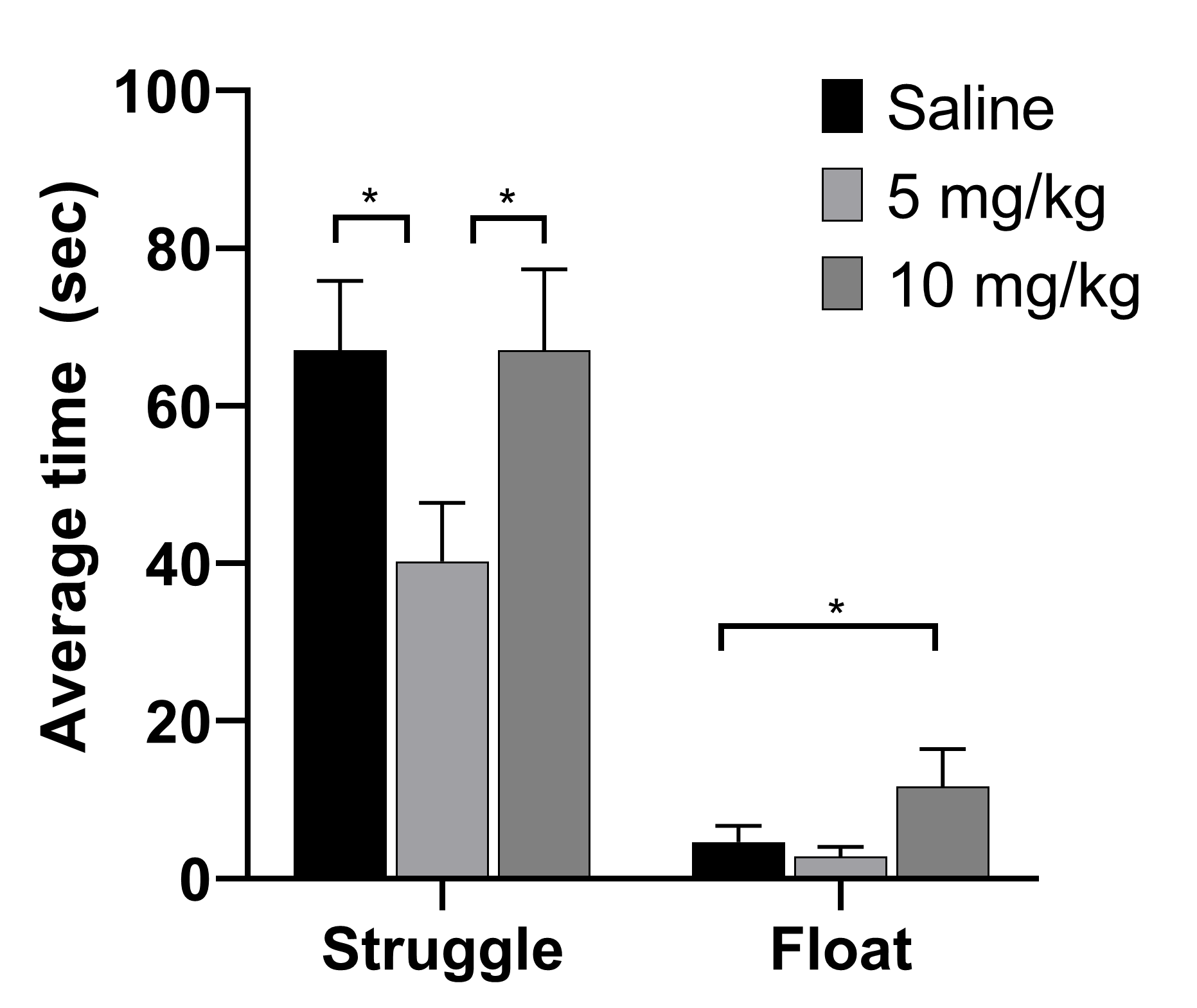

Supplement: Supplementary file 1 [file Image_1.TIF]
