# Supplementary material for: Developmental Fluoxetine Exposure Alters Behavior and Neuropeptide Receptors in the Prairie Vole
Source: Front Behav Neurosci. 2020 Nov 16;14:584731. doi: 10.3389/fnbeh.2020.584731 (PMC7701284; doi:10.3389/fnbeh.2020.584731)
Supplement: Supplementary file 2 [file Table_1.DOCX]

**Supplementary Figure 1.** Pilot dose-finding data from forced swim test in isolated adult females. At 5 mg/kg, females struggled significantly less (when compared to saline, t_1_ = -2.92, p = 0.005), and spent approximately 40% less time immobile (although this was not statistically significant). In contrast, at 10 mg/kg struggle behavior did not differ from the saline treatment, and time spent immobile (floating) trended towards an increase (when compared to saline, t_1_ = 1.64, p = 0.106).
